# A Direct Comparison of Two Densely Sampled HIV Epidemics: The UK and Switzerland

**DOI:** 10.1038/srep32251

**Published:** 2016-09-19

**Authors:** Manon L. Ragonnet-Cronin, Mohaned Shilaih, Huldrych F. Günthard, Emma B. Hodcroft, Jürg Böni, Esther Fearnhill, David Dunn, Sabine Yerly, Thomas Klimkait, Vincent Aubert, Wan-Lin Yang, Alison E. Brown, Samantha J. Lycett, Roger Kouyos, Andrew J. Leigh Brown

**Affiliations:** 1 University of Edinburgh, Edinburgh, UK; 2 Division of Infectious Diseases and Hospital Epidemiology, University Hospital Zurich, University of Zurich, Zurich, Switzerland; 3 Institute of Medical Virology, University of Zurich, Zurich, Switzerland; 4 MRC Clinical Trials Unit, London, UK; 5 Laboratory of Virology and AIDS Center, Geneva University Hospital, Geneva, Switzerland; 6 Department Biomedicine-Petersplatz, University of Basel, Basel, Switzerland; 7 Division of Immunology and Allergy, Centre Hospitalier Universitaire Vaudois and University of Lausanne, Lausanne, Switzerland; 8 Public Health England, London, UK

## Abstract

Phylogenetic clustering approaches can elucidate HIV transmission dynamics. Comparisons across countries are essential for evaluating public health policies. Here, we used a standardised approach to compare the UK HIV Drug Resistance Database and the Swiss HIV Cohort Study while maintaining data-protection requirements. Clusters were identified in subtype A1, B and C *pol* phylogenies. We generated degree distributions for each risk group and compared distributions between countries using Kolmogorov-Smirnov (KS) tests, Degree Distribution Quantification and Comparison (DDQC) and bootstrapping. We used logistic regression to predict cluster membership based on country, sampling date, risk group, ethnicity and sex. We analysed >8,000 Swiss and >30,000 UK subtype B sequences. At 4.5% genetic distance, the UK was more clustered and MSM and heterosexual degree distributions differed significantly by the KS test. The KS test is sensitive to variation in network scale, and jackknifing the UK MSM dataset to the size of the Swiss dataset removed the difference. Only heterosexuals varied based on the DDQC, due to UK male heterosexuals who clustered exclusively with MSM. Their removal eliminated this difference. In conclusion, the UK and Swiss HIV epidemics have similar underlying dynamics and observed differences in clustering are mainly due to different population sizes.

Despite huge advances in prevention and treatment, the global burden of HIV continues to rise and novel approaches are required to eliminate the virus. Phylogenetic analyses of HIV genetic sequences enable detailed reconstructions of historical and current HIV transmission. As such, phylogenetics can help identify populations at risk and better tailor public health interventions.

The UK and Switzerland have two of the most extensive HIV sequence databases in the world: the UK HIV Drug Resistance Database (UK HIV RDB) and the Swiss HIV Cohort Study (SHCS), which contain *pol* sequences from at least 60% of diagnosed patients within each country. These databases have allowed Switzerland and the UK to lead the way in population-level HIV research, including HIV phylogenetics. Phylogenetic analyses of HIV in these densely sampled epidemics have been used to estimate epidemic parameters including times between transmissions according to risk group^1^,^2^, crossover between risk groups^3^,^4^,^5^ and the relative contributions of local versus imported infections^5^.

HIV was introduced into UK men who have sex with men (MSM) around 1980^6^, probably from the USA^7^. Until the early 1990 s the epidemic was dominated by the transmission of subtype B among MSM and people who inject drugs (PWID). Six large reconstructed MSM transmission chains reflect independent introductions. The MSM epidemic displayed exponential growth in the 1980 s then stabilised in the 1990 s but continues to be highly clustered^8^. In the 1990 s, cases among heterosexuals became more common^9^ and non-B subtypes increased^10^,^11^. Within MSM clusters, 25% of transmissions occur within 6 months of infection^2^. Heterosexuals in the UK display far less clustering and slower epidemic dynamics: only 2% of transmissions occur within 6 months of infection^1^. Currently, 100,000 people are living with HIV (0.15% prevalence), one quarter of whom are unaware of their infection^12^. Highly Active Antiretroviral Therapy (HAART) became available to residents in 1996 and to all in 2012. HIV positive people on successful treatment in the UK have normal life expectancy^13^.

Switzerland had the highest HIV prevalence in Europe in the 1980 s^14^. HIV initially spread among MSM and PWID^15^ with heterosexual transmission starting to play a role after the mid-1980 s. The number of new diagnoses declined in the 1990 s, owing to needle exchange, heightened awareness, wide-scale testing, and the introduction of HAART in 1996. The number of new HIV diagnoses in Switzerland has fluctuated since 2000 with no clear time trends. The MSM and PWID epidemics display limited overlap. The heterosexual subtype B epidemic appears to be reseeded by PWID, migration, and (to a limited extent) MSM, with the importance of PWID in driving new infections decreasing over time^3^,^5^,^16^ (less than 3 PWID infections per year in the last 5 years^17^). Meanwhile only 25% of non-B infections arise within Swiss-specific clusters, indicating a growing role for immigration^5^.

One important focus of phylogenetic analyses is on clusters: groups of sequences more related to each other than to the rest of the tree. Clustered sequences represent epidemiologically linked infections with short durations of time between transmissions and thus clusters represent the leading edge of the epidemic. High clustering of sequences within a country indicates rapid transmission and a bias towards within-country transmission.

Despite the two countries sharing similar epidemic histories, separate analyses have suggested different structures of the two countries’ epidemics, including distinct proportions of clustered sequences. In the UK, 24%, 40% and 22% of patients infected with HIV-1 subtypes B^17^, A1 and C^12^, respectively, cluster, whereas the numbers for Switzerland are 55%, 21% and 16%^3^,^5^. Clearly these differences arise in part due to distinct cluster definitions: the SHCS defines a cluster as ≥10 sequences supported by >80% bootstrap^3^, while the UK studies defined clusters by a genetic distance (GD) ≤4.5% and bootstrap ≥90%^2^. Variable cluster definitions are a common problem in the literature. Bootstraps from 70% to 99% are used, in combination with GD from 1.5% to 4.5%. Another reason for the disparity could be differences in sampling procedures. However it is also possible that the contact and transmission processes between Switzerland and the UK differ. Given these observed differences, it is unclear to what extent findings from one country can be applied to the other, and more importantly whether results from either can be extended to other less densely sampled European epidemics.

Because access to data from national cohorts is subject to restrictions, and because analyses have been conducted according to in-house bioinformatics pipelines, the differences between the two epidemics have never been elucidated. Here, we present an analysis conducted in parallel on the two epidemics using a standardised approach. We hypothesised that because of its geography, the Swiss epidemic might be more integrated into the European epidemic and less clustered because of unsampled links in Swiss transmission chains. Using the same cluster definition, we compared the cluster distributions (the number and sizes of clusters) of the two countries as an indicator of underlying epidemic dynamics. We determined whether clustering was affected by risk group and ethnicity and compared the degree distributions (the number of linked partners) of heterosexual, MSM and PWID to test whether differences between countries were down to any specific risk group. Finally, we tested whether the UK and Swiss epidemics intermingled with the same foreign countries through sequence analysis.

## Results

### Baseline demographics

HIV *pol* sequences were retrieved from the SHCS drug resistance database^18^ (SHCS DRDB, 2014) and from the UK HIV RDB (sequences up to mid-2013). Closely related sequences were obtained from the Los Alamos National Laboratory (LANL) database as described in the Methods. In the final analyses, the Swiss datasets contained 1,374 subtype A1 sequences (435 Swiss, 939 LANL), 15,043 B (8,390 Swiss, 6,653 LANL) and 1,571 C (419 Swiss, 1,152 LANL). The UK datasets comprised 4,421 A1 (2,507 UK, 1,914 LANL), 38,863 B (31,450 UK, 7,413 LANL) and 22,027 C (15,815 UK, 6,212 LANL). The Swiss epidemic comprised more PWID, fewer heterosexuals and more patients of White ethnicity (Table 1). These differences were in part a result of the different subtype composition across the two countries, but even within subtype B there were notable differences, with proportionally more cases among heterosexuals and PWID in Switzerland.

In both countries individuals for whom sequence and epidemiological data were available broadly matched the characteristics of the HIV diagnosed population as a whole, in terms of risk group, sex, ethnicity and age distribution^19^,^20^. Swiss sequence dates go back to 1995 owing to retrospective sequencing of samples from the SHCS Bio-bank^21^.

### Difference in clustering

**Figure 1:**
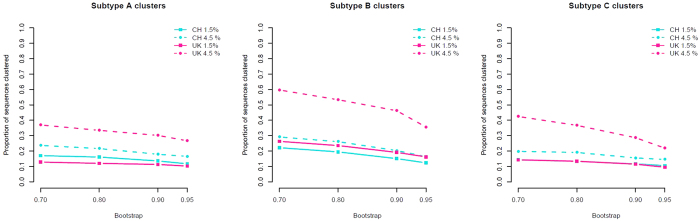
Proportion of UK (pink) and Swiss (blue) sequences in clusters at different genetic distance (1.5% and 4.5%) and bootstrap (70%, 80%, 90%, 95%) thresholds. CH - Switzerland.

Maximum likelihood phylogenetic trees were constructed and clusters were selected^22^ at a range of genetic distance (GD) and bootstrap thresholds. A significantly larger proportion of UK subtype B sequences clustered in the uncorrected analysis at all thresholds (Supplementary Table 1, Fig. 1). Concordantly, the UK was more clustered in the univariate analysis (Table 2) with odds for being in a cluster 4 times higher at 4.5% GD. At 4.5% GD, subtypes C and A1 were also more clustered in the UK, but there was no significant difference at 1.5% GD. The subsequent analysis focused mainly on subtype B.

Because of the difference in sampling time distributions and demographics between the two subtype B datasets, we considered the logistic regression adjusting for those variables. More recent samples were much more likely to cluster than older samples (Table 3) and when the model was adjusted for sample date, the Swiss epidemic was more clustered than the UK epidemic at 1.5% GD. At 4.5% GD, clustering remained higher for the UK but the strength of the association was halved compared with the univariate model. The effect of risk group and ethnicity on clustering was consistent across the two countries, with MSM in both countries showing the highest propensity for clustering.

### Degree Distributions

**Figure 2:**
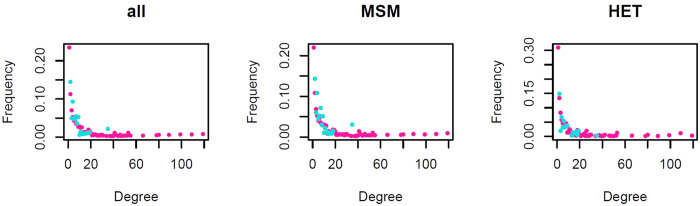
Degree distributions of the UK (pink) and Swiss (blue) subtype B epidemics. MSM - men who have sex with men, HET - heterosexuals. Cluster definition was: genetic distance = 4.5% and bootstrap = 90%. Note that the proportion of individuals of each degree is shown rather than the absolute number of individuals of each degree. The number of clustered individuals was much larger in the UK than in Switzerland.

Degree distributions for each country and risk group were generated based on cluster size distributions and compositions. For example a cluster containing 3 heterosexuals is equivalent to 3 heterosexuals each with degree 2. Statistical frameworks exist to formally compare degree distributions^23^,^24^,^25^ and include bootstrapping to simulate the effect of the sampling process and test the robustness of conclusions. Degree distributions for the populations as a whole and for each risk group were compared using the Kolmogorov-Smirnov (KS) tests^24^ and the Degree Distribution Quantification and Comparison (DDQC) algorithm^23^. Based on the KS test, there was no difference between the two countries at 1.5% GD. At 4.5% GD, distributions differed significantly for HET, MSM and the population as a whole, but not for PWID (Supplementary Table 2). The difference appeared to be driven by the longer tail of the UK distributions (Fig. 2), indicating the existence of larger clusters in the UK. The UK epidemic thus comprised not only a higher proportion of sequences in clusters, but also clusters were larger.

**Figure 3:**
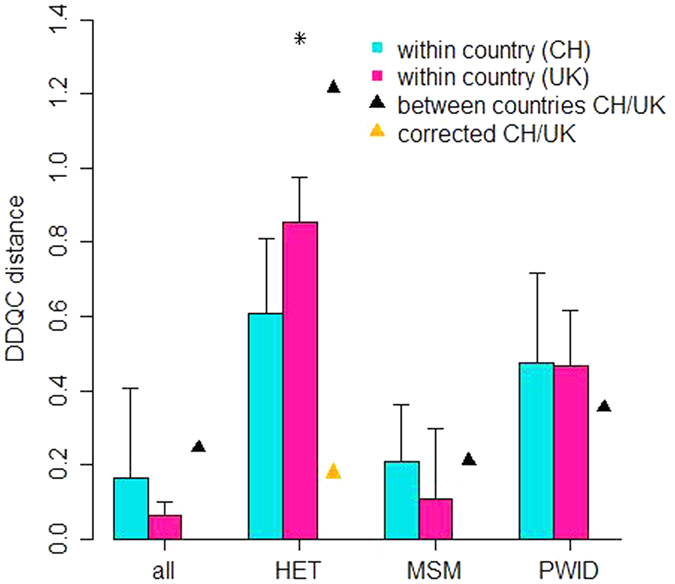
DDQC distances within and between countries. DDQC – Degree Distribution Quantification and Comparison, CH - Switzerland, MSM - men who have sex with men, HET - heterosexuals. PWID – people who inject drugs. Cluster definition was: genetic distance = 4.5% and bootstrap = 90%. In order to generate null distributions of the expected values for the DDQC, we bootstrapped the Swiss and UK distributions and calculated DDQC values comparing the original datasets to their bootstrap samples (in blue and pink). The top of the coloured bars represent the mean distance of within country comparisons and the whiskers represent the 95% percentiles. The DDQC distance was then calculated between the UK and Swiss degree distributions (black triangles). The distance between countries was considered significant if it exceeded the 95% percentile from the simulated values, which was the case only for HET at 4.5% GD (indicated by *). When we removed heterosexuals who were likely to have been infected through sex with men from the UK dataset, the DDQC distance between the UK and Swiss HET degree distributions fell within the simulated null distribution (orange triangle).

Because the KS test is sensitive to network size, we applied the DDQC, which is robust to differences in scale. The DDQC measures the distance between networks based on features extracted from their degree distributions. However, it does not indicate whether distances calculated are significant or not. In order to generate null distributions, the UK and Swiss degree distributions were compared to themselves through bootstrapping. The UK and Swiss degree distributions were each bootstrapped 100 times to simulate the effect of sampling (see Methods) and the DDQC distance calculated between the true data and each bootstrap replicate (Fig. 3). Between country DDQC values were considered significant if they exceeded the 95% percentile of the within country DDQC values (one-sided test). The DDQC distance was higher than expected only for heterosexuals at 4.5% GD (95^th^ percentile DDQC = 0.97, observed HET DDQC = 1.22).

**Figure 4:**
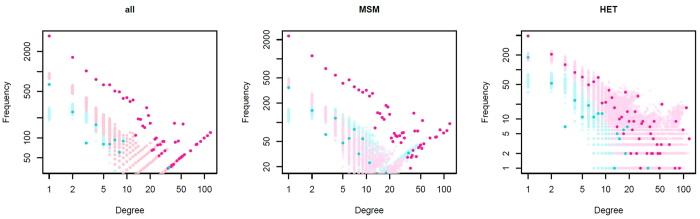
Jack-knife and bootstrap sampled degree distributions of the UK (pink) and Swiss (blue) epidemics. UK subtype B degree distribution for men who have sex with men (MSM), heterosexuals (HET) and the population as a whole (all) were jack-knife sampled 100 times to match the size of the Swiss epidemics (in light pink). The Swiss epidemic was bootstrapped 100 times to its full size (in light blue). Degree distributions are shown on a double-logged scale. Samples overlapped for MSM and the dataset as a whole, but not for HET. Where Swiss replicates cannot be seen they are covered by the UK replicates.

We hypothesised that the difference highlighted by the KS test at 4.5% might be the result of a difference in scale between the two epidemics. The UK population (and the HIV+ population) is much larger than that of Switzerland and so the pool of partners available is bigger. To examine the effect of epidemic size on clustering and degree distributions, we down-sampled the UK subtype B datasets to match the size of the Swiss datasets. In parallel, the Swiss datasets were bootstrap sampled with replacement. When these equal-sized resampled datasets were compared, the UK and Swiss degree distributions overlapped for the population as a whole and for the MSM population, but not for heterosexuals (Fig. 4).

In the true and the jack-knife sampled UK heterosexual population, we observed male heterosexuals with high degree (>20) not present in the Swiss data (Fig. 4). The largest exclusively heterosexual cluster in the UK comprised 27 individuals (bootstrap = 0.9, GD = 4.5%); all heterosexuals with higher degree were in clusters dominated by MSM. When we dropped heterosexuals with degree >26 from the UK sample (125 individuals out of a total of 1556 heterosexuals), the DDQC distance between the two networks fell within its null distribution (DDQC = 0.17, Fig. 3).

### Cross border transmission

We investigated intermingling between national and foreign sequences by removing the 80% national criterion. We used a tight GD threshold (1.5%, 70% bootstrap) to capture close transmission partners. At this threshold, Swiss sequences clustered with 162 non-Swiss sequences and UK sequences clustered with 353 non-UK sequences. For Switzerland, Western European countries provided over 75% of the links. For the UK, 50% of close links were with other European countries and 20% originated from other Anglophone countries: Australia, Canada and the USA (Supplementary Figure 1).

## Discussion

The aim of this study was to compare epidemic dynamics between the two most densely sampled HIV epidemics, the UK and Switzerland, while adhering to data governance procedures and privacy protection requirements. We found that the fraction of sequences in transmission clusters was similar between the UK and Switzerland for a strict GD threshold (1.5%) but that they differed at a more relaxed GD threshold (4.5%). This suggests that the two epidemics resemble each other at a micro-level but differ at a macro-level. Because a statistical framework for comparing cluster distributions directly is lacking, we generated degree distributions based on cluster sizes and compared them through formal statistical tests: the KS test, the DDQC and bootstrapping. Based on the KS test, there were differences between the UK and Swiss subtype B degree distributions at 4.5% GD. However, downsampling the UK dataset to the size of the Swiss dataset rendered this difference insignificant in MSM and the population as a whole, but not heterosexuals (Fig. 4). In parallel, only heterosexuals showed a significant difference based on the DDQC test, which corrects for network size.

The degree distribution of UK heterosexuals had a long tail representing male heterosexuals clustered exclusively with MSM. Previous UK analyses have demonstrated that a proportion of self-reported male heterosexuals are likely to have been infected through sex with men^26^, which appears to be the case here. When those high degree heterosexuals were removed from the dataset, the UK and Swiss no longer differed. Male heterosexuals who have sex with men are likely to also have sex with women and provide a bridge between MSM and heterosexual epidemics. This is a likely route for the spread of non-B subtypes among MSM in the UK^4^. More detailed analyses of the Swiss epidemic have found little overlap between the MSM and HET epidemics^3^; however, the Swiss heterosexual with the highest degree was similarly part of a HET/MSM cluster comprising 36 individuals, while the largest exclusively heterosexual cluster contained only 9. In fact, 47% of UK and 38% of Swiss heterosexuals were in HET/MSM clusters. The present analysis cannot determine whether bridging is more common in the UK or whether risk group classification is assessed more thoroughly in Switzerland.

There was more overlap between PWID and heterosexuals in Switzerland than in the UK (23% vs 12%), but the difference in PWID degree distributions was not significant. Although this could be due to sample size, the stemming of the heterosexual epidemic through PWID in Switzerland is likely to be an old process^3^ while the bridging between HET and MSM in the UK is ongoing^4^,^26^.

Our findings were consistent across bootstrap thresholds. At 1.5% GD, we found no difference between the degree distributions of the Swiss and UK epidemics. At tight thresholds mostly pairs and recently infected patients are captured and these groupings are similar across the two countries. At 4.5% GD, the UK was more clustered and so the UK HIV RDB is more likely to capture larger transmission chains. However, the downsampled UK epidemic degree distribution overlapped with the Swiss degree distribution. While the proportion of clustered individuals in the UK is higher, the difference is seemingly due to the UK greater epidemic size rather than because of differences in contact or transmission processes. Both countries are similarly integrated into global unsampled epidemics, and this study underlines the importance of HIV public health interventions at the European and global levels.

Transmission between European countries has been analysed in more depth elsewhere^27^. In agreement with that analysis, we found Spain to be a major mixing partner for both Switzerland and the UK. Germany and the Czech Republic were also identified as significant. We found increased linkage between the UK and other Anglophone countries. In Switzerland strong segregation has been observed between German and French-speaking regions^3^ and this language-dependency of HIV transmission warrants investigation at the global scale.

Both countries have noted the subtype diversification of their respective epidemics^9^,^28^, yet the difference in size between the UK and Swiss subtype A1 and C datasets (18,000 vs 900, respectively) rendered a comparison meaningless. In Switzerland fewer than 25% of non-B infections were acquired in the country^5^, whereas in the UK over 50% of infections in individuals born abroad are thought to have occurred in the UK^11^. Local non-B non-heterosexual transmission appears far more extensive in the UK^4^.

Although degree distributions are a blunt tool for elucidating the dynamics of an epidemic^29^, they allowed us to apply statistically robust methods to compare the two epidemics without the need for exchanging sensitive data. Sequences from national databases were never exchanged and while this precluded a combined phylogenetic analysis of UK and Swiss sequences, one of the strengths of the study stems from undertaking such an analysis without compromising patient privacy. A second issue, the distributions of sample dates differing between the two cohorts, arose because the SHCS has conducted extensive retrospective sequencing on patients diagnosed early on in the epidemic and for whom samples had been stored. The SHCS coverage of older samples explains in part the lower clustering observed in Switzerland. However, clustering remained significantly higher in the UK at 4.5% GD after sample date was adjusted for. Thirdly, international comparisons were based on the LANL database which is in essence a large-scale convenience sample and not necessarily representative. We suggest the apparently important contribution of the Czech Republic to both epidemics may arise from recent submission of large numbers of sequences from that country.

In conclusion, we showed that apparent major differences in clustering patterns between the UK and Switzerland subtype B epidemics can be explained for the most part by differences in size and sampling time. This is the first study leveraging the vast amounts of data available in multiple national HIV databases. We made use of data without breaching data governance procedures and highlighted that transmission trends in these two countries are driven by similar underlying factors. From a methodological perspective, our study highlights the importance of using the same cluster-detection algorithm and correcting for demographic factors when comparing clustering patterns across settings.

## Methods

### Data

#### Switzerland

9,232 HIV *pol* sequences were retrieved from the SHCS DRDB. The SHCS DRDB aggregates all HIV resistance tests for patients of the SHCS. SmartGene is responsible for data storage and management (http://www.smartgene.com). The DRDB is part of the SHCS, which is an ongoing national clinical cohort of HIV patients aged 16 and above with biannual follow up (http://www.shcs.ch)^20^. Sequences were assigned subtypes using REGA^30^,^31^; subtypes B (91%), A1 (5%), and C (5%) were analysed. The SHCS has been approved by the ethics committees of all participating institutions, and written, informed consent has been obtained from participants.

#### UK

63,065 HIV *pol* sequences were obtained from the UK HIV RDB (http://www.hivrdb.org). Sequences cover protease and 900 bases of reverse transcriptase. After age, sex, risk group and ethnicity were attached, the data were anonymised and delinked. Subtypes were assigned using SCUEAL^32^ and REGA^30^,^31^. The three most common subtypes were B (48.6%), C (24.4%), and A1 (4.0%). Ethical approval was given by the London Multicentre Research Ethics Committee (MREC/01/2/10; 5 April 2001).

As submission of the UK and Swiss sequence datasets to public databases would permit transmission network identification and thus risk breaching patient confidentiality, we have followed earlier practice^3^,^33^. A random sample of 10% of each subtype and country has been submitted to Genbank (accession numbers available in supplementary material).

#### Background sequences

All *pol* (HXB2 positions 2253–3870) sequences of HIV subtype A1, B, and C longer than 900 bases were retrieved from LANL (January 2014). To limit the size of alignments, the ten closest sequences to each of the local (UK and Swiss) sequences were selected using Viroblast^34^. For this step, UK sequences were removed from LANL alignments before the UK Viroblast run, and Swiss LANL sequences were removed before the Swiss run.

Only the earliest available sequence for each individual was used. All sequences were stripped of 44 sites associated with drug resistance based on the 2013 International AIDS Society list^35^.

### Tree Building and Cluster Picking

Duplicate sequences were removed. Maximum likelihood phylogenetic trees were constructed for each country and subtype separately (six trees in total) using FastTree v2.0^36^ with 100 bootstraps. Initially clusters were selected for further analysis if they were supported by bootstrap thresholds of 70%, 80%, 90% and 95% and maximum GD of 1.5% or 4.5% (8 thresholds total)^22^. Of the initially identified clusters, those in the Swiss trees were further selected to contain at least 80% SHCS sequences, and clusters in the UK trees at least 80% UK sequences. In a separate analysis, we examined all clusters with at least one UK or Swiss sequence (within the respective datasets) to investigate mixing between national and foreign sequences. The automated pipeline included analysis with the Cluster Picker and Cluster Matcher^22^ as well as processing through python and R scripts (available upon request). The Cluster Picker was upgraded to recognise IUPAC nucleotide ambiguity codes as matches (version available upon request from the authors), increasing clustering by around 15% in both datasets.

From the Cluster Picker and Cluster Matcher output files, we generated degree distributions (the number of links for each node). As files contained risk group composition for each cluster, it was possible to break down degree distribution by risk group. Nodes were sampled with replacement from the network with information on their cluster membership. Nodes sampled with the same cluster membership were linked together in each bootstrapped network, so that clusters sometimes increased in size, sometimes decreased in size or otherwise disappeared, and degree distribution was re-estimated each time. Jack-knife resampling where the number of nodes sampled was smaller than the full network size was also performed.

### Statistical analysis

The number of sequences clustering at different thresholds between the two epidemics was compared using Fisher’s exact test with Bonferroni correction (24 comparisons across clustering thresholds and subtypes). Degree distributions were compared using the KS test^24^ and the DDQC algorithm^23^. The KS test is a nonparametric test which compares the cumulative distribution of two samples to estimate whether they have been drawn from the same distribution and is frequently used to compare degree distributions^25^. The DDQC was developed specifically to compare degree distributions and corrects for differences in population size while the KS test does not. The DDQC extracts a vector of eight values from degree distributions for comparison. In brief, the range of node degrees is divided into eight regions based on the minimum, maximum, mean and standard deviation of the degree distribution. The probability of the degree of any node being contained within each interval is calculated. The distance between two networks is the sum of the absolute differences for each of the eight features extracted.

The UK subtype B dataset used here was 3.75 times larger than the Swiss dataset; the UK MSM dataset was 5.7 times larger and the UK heterosexual dataset was 1.3 times larger (Table 1). To investigate the effect of the difference in size of the pool of possible infectors, the UK dataset was jack-knife sampled to the size of the Swiss dataset. One hundred jack-knife replicates were generated, and in each replicate the degree distribution was re-estimated based on the links present in the sample.

A logistic regression model was used to characterise the factors influencing clustering in the two countries. The model was applied with cluster membership as the outcome variable and with the country of origin (UK or Switzerland) as the main exposure variable. Sampling dates, risk group, sex, and ethnicity were adjusted for. Statistical analyses were conducted in R^37^.

## Additional Information

**How to cite this article**: Ragonnet-Cronin, M. L. *et al*. A Direct Comparison of Two Densely Sampled HIV Epidemics: The UK and Switzerland. *Sci. Rep.* **6**, 32251; doi: 10.1038/srep32251 (2016).

## Supplementary Material

Supplementary Information

## Figures and Tables

**Table 1:** Baseline demographics of the two datasets.

Sex	Female	UK			CH		
		A1	B	C	A1	B	C
		1442 (57.5%)	2327 (7.4%)	3869 (62.2%)	269 (62%)	1812 (22%)	237 (57%)
Risk	MSM	170 (6.8%)	22157 (70.4%)	660 (4.2%)	21 (5%)	3914 (47%)	25 (6%)
	HET	1718 (68.5%)	3399 (10.8%)	11893 (75.2%)	366 (84%)	2626 (31%)	357 (85%)
	PWID	92 (3.7%)	873 (2.8%)	128 (0.8%)	12 (3%)	1553 (19%)	9 (2%)
	Other/NA	527 (21%)	5021 (16%)	3134 (19.8%)	36 (8%)	287 (3%)	28 (7%)
Ethnicity	White	494 (19.7%)	22724 (72.3%)	1724 (10.9%)	194 (45%)	7333 (87%)	118 (28%)
Total		2507	31450	15815	435	8390	419

**Table 2:** Unadjusted logistic regression for the comparison of the degree of patients clustering between the UK and Switzerland (Subtype B).

Bootstrap	Genetic Distance	Covariates	OR	2.5%	97.5%
0.7	0.015	UK	1.316	1.243	1.394
0.8	0.015	UK	1.335	1.258	1.418
0.9	0.015	UK	1.396	1.308	1.492
0.95	0.015	UK	1.444	1.345	1.551
0.7	0.045	UK	5.001	4.745	5.272
0.8	0.045	UK	4.119	3.904	4.347
0.9	0.045	UK	3.942	3.722	4.176
0.95	0.045	UK	3.176	2.986	3.381

OR: odds-ratio.

**Table 3:** Logistic regression for the comparison of the degree of patients clustering between the UK and Switzerland, corrected for sample year (Subtype B).

Bootstrap	Genetic Distance	Covariates	OR	2.5%	97.5%
0.7	0.015	UK	0.66	0.617	0.705
		Sample year	1.138	1.131	1.145
0.8	0.015	UK	0.665	0.621	0.713
		Sample year	1.142	1.134	1.149
0.9	0.015	UK	0.694	0.645	0.749
		Sample year	1.146	1.138	1.154
0.95	0.015	UK	0.706	0.651	0.765
		Sample year	1.153	1.144	1.162
0.7	0.045	UK	2.692	2.538	2.856
		Sample year	1.134	1.128	1.14
0.8	0.045	UK	2.12	1.996	2.252
		Sample year	1.145	1.139	1.151
0.9	0.045	UK	1.968	1.846	2.099
		Sample year	1.156	1.15	1.163
0.95	0.045	UK	1.551	1.448	1.662
		Sample year	1.161	1.154	1.168

OR: odds-ratio.

## References

[b1] Hughes G. J. . Molecular phylodynamics of the heterosexual HIV epidemic in the United Kingdom. PLoS Pathog. 5(9), e1000590 (2009). 19779560 10.1371/journal.ppat.1000590 PMC2742734

[b2] Lewis F., Hughes G. J., Rambaut A., Pozniak A. & Leigh Brown A. J. Episodic sexual transmission of HIV revealed by molecular phylodynamics. PLoS. Med. 5(3), e50 (2008). 18351795 10.1371/journal.pmed.0050050 PMC2267814

[b3] Kouyos R. D. . Molecular epidemiology reveals long-term changes in HIV type 1 subtype B transmission in Switzerland. J Infect Dis 201(10), 1488 (2010). 20384495 10.1086/651951

[b4] Ragonnet-Cronin M. . Transmission of Non-B HIV Subtypes in the United Kingdom Is Increasingly Driven by Large Non-Heterosexual Transmission Clusters. J. Infect. Dis. 213(9), 1410 (2016). 26704616 10.1093/infdis/jiv758 PMC4813743

[b5] von Wyl V. . The role of migration and domestic transmission in the spread of HIV-1 non-B subtypes in Switzerland. J. Infect. Dis. 204(7), 1095 (2011). 21881125 10.1093/infdis/jir491

[b6] Pinching A. J. . Studies of cellular immunity in male homosexuals in London. Lancet 2(8342), 126 (1983). 6134980 10.1016/s0140-6736(83)90115-0

[b7] Thomson M. M. & Najera R. Increasing HIV-1 genetic diversity in Europe. J. Infect. Dis. 196(8), 1120 (2007). 17955428 10.1086/521683

[b8] Hue S., Pillay D., Clewley J. P. & Pybus O. G. Genetic analysis reveals the complex structure of HIV-1 transmission within defined risk groups. Proc. Natl. Acad. Sci. USA 102(12), 4425 (2005). 15767575 10.1073/pnas.0407534102 PMC555492

[b9] The UK Collaborative Group on HIV Drug Resistance The increasing genetic diversity of HIV-1 in the UK, 2002–2010. AIDS 28(5), 773 (2014). 24257094 10.1097/QAD.0000000000000119 PMC3940288

[b10] Aggarwal I. . Evidence for onward transmission of HIV-1 non-B subtype strains in the United Kingdom. J. Acquir. Immune. Defic. Syndr. 41(2), 201 (2006). 16394853 10.1097/01.qai.0000179430.34660.11

[b11] Rice B. D., Elford J., Yin Z. & Delpech V. C. A new method to assign country of HIV infection among heterosexuals born abroad and diagnosed with HIV. AIDS 26(15), 1961 (2012). 22781226 10.1097/QAD.0b013e3283578b80

[b12] Yin Z. . HIV in the United Kingdom: 2014 Report. [Report] (2014).

[b13] May M. T. . Impact on life expectancy of HIV-1 positive individuals of CD4+ cell count and viral load response to antiretroviral therapy. AIDS 28(8), 1193 (2014). 24556869 10.1097/QAD.0000000000000243 PMC4004637

[b14] EuroHIV: European Centre for the Epidemiological Monitoring of AIDS & UNAIDS. HIV/AIDS Surveillance in Europe [Report] (1999). 1462717

[b15] Csete J. & Grob P. J. Switzerland, HIV and the power of pragmatism: lessons for drug policy development. Int. J. Drug Policy 23(1), 82 (2012). 21852097 10.1016/j.drugpo.2011.07.011

[b16] Stadler T. . Estimating the basic reproductive number from viral sequence data. Mol. Biol. Evol. 29(1), 347 (2012). 21890480 10.1093/molbev/msr217

[b17] Nationales Programm HIV und ande-re sexuell übertragbare Infektionen 2011–2017 (NPHS). HIV- und STI-Fallzahlen 2012: Berichterstattung, Analysen und Trends [Report] (2013).

[b18] von Wyl V. . Emergence of HIV-1 drug resistance in previously untreated patients initiating combination antiretroviral treatment: a comparison of different regimen types. Arch. Intern. Med. 167(16), 1782 (2007). 17846398 10.1001/archinte.167.16.1782

[b19] Aghaizu A., Brown A. E., Nardone A., Gill O. N. & Delpech V. HIV in the United Kingdom: 2013 Report. [Report] (2013).

[b20] Schoeni-Affolter F. . Cohort profile: the Swiss HIV Cohort study. Int. J. Epidemiol. 39(5), 1179 (2010). 19948780 10.1093/ije/dyp321

[b21] Yang W. L. . Assessing the Paradox Between Transmitted and Acquired HIV Type 1 Drug Resistance Mutations in the Swiss HIV Cohort Study From 1998 to 2012. J. Infect. Dis. 212(1), 28 (2015). 25576600 10.1093/infdis/jiv012

[b22] Ragonnet-Cronin M. . Automated analysis of phylogenetic clusters. BMC. Bioinformatics 14(1), 317 (2013). 24191891 10.1186/1471-2105-14-317 PMC4228337

[b23] Aliakbary S., Habibi J. & Movaghar A. Feature Extraction from Degree Distribution for Comparison and Analysis of Complex Networks. Comput. J. 12 (2015).

[b24] Massey F. J. The Kolmogorov-Smirnov Test for Goodness of Fit. Journal of the American Statistical Association 46(253), 68 (1951).

[b25] Wang X., Latapy M. & Soria M. Deciding on the type of the degree distribution of a graph from traceroute-like measurements. International Journal of Computer Networks & Communications 4(3), 151 (2012).

[b26] Hue S. . Phylogenetic analyses reveal HIV-1 infections between men misclassified as heterosexual transmissions. AIDS 28(13), 1967 (2014). 24991999 10.1097/QAD.0000000000000383

[b27] Paraskevis D. . Tracing the HIV-1 subtype B mobility in Europe: a phylogeographic approach. Retrovirology. 6, 49 (2009). 19457244 10.1186/1742-4690-6-49 PMC2717046

[b28] Neogi U. . Temporal trends in the Swedish HIV-1 epidemic: increase in non-B subtypes and recombinant forms over three decades. PLoS. One. 9(6), e99390 (2014). 24922326 10.1371/journal.pone.0099390 PMC4055746

[b29] Hamilton D. T., Handcock M. S. & Morris M. Degree distributions in sexual networks: a framework for evaluating evidence. Sex Transm. Dis. 35(1), 30 (2008). 18217224 10.1097/olq.0b013e3181453a84 PMC4370286

[b30] Alcantara L. C. . A standardized framework for accurate, high-throughput genotyping of recombinant and non-recombinant viral sequences. Nucleic Acids Res. 37, (Web Server issue), W634–W642 (2009). 19483099 10.1093/nar/gkp455 PMC2703899

[b31] de Oliveira T. . An automated genotyping system for analysis of HIV-1 and other microbial sequences. Bioinformatics. 21(19), 3797 (2005). 16076886 10.1093/bioinformatics/bti607

[b32] Kosakovsky Pond S. L. . An evolutionary model-based algorithm for accurate phylogenetic breakpoint mapping and subtype prediction in HIV-1. PLoS. Comput. Biol. 5(11), e1000581 (2009). 19956739 10.1371/journal.pcbi.1000581 PMC2776870

[b33] Leigh Brown A. J. . Transmission network parameters estimated from HIV sequences for a nationwide epidemic. J. Infect. Dis. 204(9), 1463 (2011). 21921202 10.1093/infdis/jir550 PMC3182313

[b34] Deng W., Nickle D. C., Learn G. H., Maust B. & Mullins J. I. ViroBLAST: a stand-alone BLAST web server for flexible queries of multiple databases and user’s datasets. Bioinformatics. 23(17), 2334 (2007). 17586542 10.1093/bioinformatics/btm331

[b35] Johnson V. A. . Update of the drug resistance mutations in HIV-1: March 2013. *Top*. Antivir. Med. 21(1), 6 (2013). PMC6148891 23596273

[b36] Price M. N., Dehal P. S. & Arkin A. P. FastTree 2–approximately maximum-likelihood trees for large alignments. PLoS One 5(3), e9490 (2010). 20224823 10.1371/journal.pone.0009490 PMC2835736

[b37] R Development Core Team. R: A language and environment for statistical computing (R Foundation for Statistical Computing, Vienna, Austria, 2011).

