# Supplementary material for: A Direct Comparison of Two Densely Sampled HIV Epidemics: The UK and Switzerland
Source: Sci Rep. 2016 Sep 19;6:32251. doi: 10.1038/srep32251 (PMC5027562; doi:10.1038/srep32251)
Supplement: Supplementary Information [file srep32251-s1.pdf]

A DIRECT COMPARISON OF TWO DENSELY SAMPLED HIV EPIDEMICS: THE UK AND SWITZERLAND.

Manon L. Ragonnet-Cronin[1]*, Mohaned Shilaih[2,3]*, Huldrych Günthard[2,3], Emma B. Hodcroft[1], Jürg Böni[3], Esther Fearnhill[4], David Dunn[4], Sabine Yerly[5], Thomas Klimkait[6], Vincent Aubert[7], Wan-Lin Yang[2], Alison E. Brown[8], Samantha Lycett[1], Roger Kouyos[2,3*] and Andrew J. Leigh Brown[1]*.

* equal contribution

1. University of Edinburgh, Edinburgh, UK; 2. Division of Infectious Diseases and Hospital Epidemiology, University Hospital Zurich, University of Zurich, Zurich, Switzerland; 3. Institute of Medical Virology, University of Zurich, Zurich, Switzerland; 4. MRC Clinical Trials Unit, London, UK; 5. Laboratory of Virology and AIDS Center, Geneva University Hospital, Geneva, Switzerland; 6. Department Biomedicine-Petersplatz, University of Basel, Basel, Switzerland; 7. Division of Immunology and Allergy, Centre Hospitalier Universitaire Vaudois and University of Lausanne, Lausanne, Switzerland; 8. Public Health England, London, UK

Supplementary material

Supplementary Table 1: Proportion of sequences clustering at different cluster thresholds for the UK and Switzerland subtypes A1, B and C.

Subtype	Bootstrap	Genetic Distance	Proportion clustered (CH)	Proportion clustered (UK)	p.Bonferroni
**A1**	0.7	0.015	0.17	0.13	0.53
**A1**	0.8	0.015	0.16	0.12	0.55
**A1**	0.9	0.015	0.14	0.11	1
**A1**	0.95	0.015	0.12	0.1	1
**A1**	0.7	0.045	0.24	0.37	<0.0001
**A1**	0.8	0.045	0.22	0.33	<0.0001
**A1**	0.9	0.045	0.18	0.3	<0.0001
**A1**	0.95	0.045	0.17	0.27	<0.0001
**B**	0.7	0.015	0.22	0.26	<0.0001
**B**	0.8	0.015	0.19	0.24	<0.0001
**B**	0.9	0.015	0.15	0.19	<0.0001
**B**	0.95	0.015	0.12	0.16	<0.0001
**B**	0.7	0.045	0.29	0.6	<0.0001
**B**	0.8	0.045	0.26	0.53	<0.0001
**B**	0.9	0.045	0.2	0.46	<0.0001
**B**	0.95	0.045	0.16	0.36	<0.0001
**C**	0.7	0.015	0.14	0.14	1
**C**	0.8	0.015	0.13	0.13	1
**C**	0.9	0.015	0.12	0.11	1
**C**	0.95	0.015	0.11	0.09	1
**C**	0.7	0.045	0.2	0.42	<0.0001
**C**	0.8	0.045	0.19	0.37	<0.0001
**C**	0.9	0.045	0.16	0.29	<0.0001
**C**	0.95	0.045	0.15	0.22	<0.001

Note: p-values shown are from Fisher's exact test, Bonferroni- corrected for multiple comparisons

Supplementary Table 2: Results of the KS test for comparing degree distributions between the UK and Switzerland (Bonferroni-corrected)

Bootstrap	Genetic Distance	Risk group	p.Bonferroni
0.9	0.045	all	0
0.9	0.045	MSM	0
0.9	0.045	HET	<0.0001
0.9	0.045	PWID	1

Note: p values are shown only for bootstrap=0.9 but significance was consistent across bootstraps

Supplementary Figure 1: Origin of close linkages for Switzerland (left) and the UK (right)

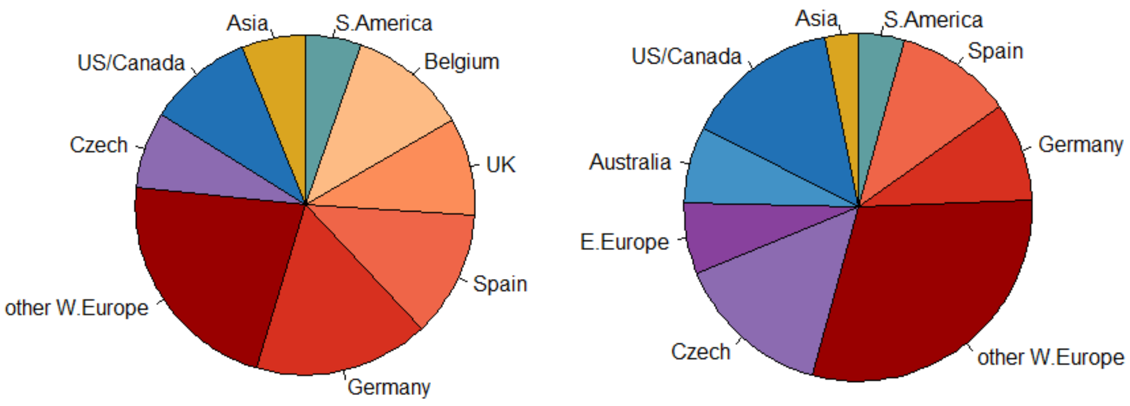

Swiss subtype B sequences clustered closely (1.5% genetic distance, 70% bootstrap) with 162 non-Swiss sequences and UK sequences clustered with 353.

Genbank accession codes

UK

KU498303 - KU499301, EU236439 - EU236538, JN100661 - JN101948, KX661407 - KX663330, Q462040, Q462050, Q462058, Q462067, Q462088, Q462094, Q462095, Q462096, Q462098, Q462100, Q462107, Q462109, Q462129, Q462141, Q462144, Q462156, Q462157, Q462159, Q462164, Q462166, Q462172, Q462175, Q462183, Q462184, Q462216, Q462226, Q462231, Q462234, Q462260, Q462264, Q462267, Q462272, Q462300, Q462333, Q462335, Q462353, Q462403, Q462413, Q462416, Q462417, Q462446, Q462463, Q462464, Q462501, Q462526, Q462532, Q462027, Q462029, Q462030, Q462031, Q462032, Q462033, Q462034, Q462036, Q462042, Q462046, Q462047, Q462049, Q462054, Q462057, Q462059, Q462062, Q462064, Q462070, Q462071, Q462072, Q462074, Q462075, Q462077, Q462081, Q462082, Q462083, Q462085, Q462086, Q462087, Q462089, Q462090, Q462091, Q462093, Q462097, Q462101, Q462102, Q462103, Q462111, Q462112, Q462113, Q462116, Q462118, Q462120, Q462121, Q462123, Q462124, Q462126, Q462128, Q462134, Q462137, Q462139, Q462143, Q462147, Q462148, Q462149, Q462150, Q462152, Q462153, Q462154, Q462155, Q462158, Q462161, Q462163, Q462167, Q462171, Q462173, Q462176, Q462178, Q462179, Q462182, Q462185, Q462186, Q462187, Q462189, Q462195, Q462196, Q462197, Q462198, Q462199, Q462200, Q462208, Q462209, Q462212, Q462213, Q462219, Q462222, Q462227, Q462228, Q462229, Q462230, Q462235, Q462239, Q462240, Q462241, Q462242, Q462244, Q462245, Q462247, Q462248, Q462254, Q462262, Q462265, Q462266, Q462269, Q462273, Q462274, Q462278, Q462280, Q462282, Q462283, Q462285, Q462290, Q462291, Q462294, Q462295, Q462299, Q462301, Q462302, Q462305, Q462306, Q462308, Q462309, Q462311, Q462312, Q462314, Q462315, Q462316, Q462319, Q462320, Q462321, Q462322, Q462325, Q462326, Q462327, Q462328, Q462329, Q462330, Q462331, Q462332, Q462334, Q462337, Q462338, Q462341, Q462342, Q462343, Q462344, Q462346, Q462347, Q462348, Q462349, Q462350, Q462351, Q462352, Q462356, Q462357, Q462358, Q462359, Q462363, Q462364, Q462366, Q462369, Q462371, Q462372, Q462374, Q462375, Q462376, Q462377, Q462382, Q462383, Q462387, Q462392, Q462393, Q462394, Q462395, Q462396, Q462397, Q462398, Q462399, Q462400, Q462401, Q462404, Q462406, Q462407, Q462411, Q462412, Q462414, Q462415, Q462418, Q462419, Q462423, Q462424, Q462425, Q462426, Q462428, Q462441, Q462443, Q462444, Q462447, Q462448, Q462450, Q462454, Q462455, Q462457, Q462458, Q462460, Q462461, Q462466, Q462467, Q462468, Q462473, Q462474, Q462476, Q462477, Q462481, Q462482, Q462483, Q462484, Q462485, Q462486, Q462487, Q462488, Q462489, Q462491, Q462496, Q462497, Q462498, Q462500, Q462502, Q462503, Q462504, Q462505, Q462506, Q462507, Q462508, Q462509, Q462510, Q462511, Q462514, Q462515, Q462516, Q462518, Q462521, Q462522, Q462523, Q462524, Q462527, Q462529, Q462530, Q462531

Switzerland

GU344102-GU344671

KX691913 - KX692269

Steering committee members

Members of the Swiss HIV Cohort Study

Aubert V, Battegay M, Bernasconi E, Böni J, Bucher HC, Burton-Jeangros C, Calmy A, Cavassini M, Dollenmaier G, Egger M, Elzi L, Fehr J, Fellay J, Furrer H (Chairman of the Clinical and Laboratory Committee), Fux CA, Gorgievski M, Günthard H (President of the SHCS), Haerry D (deputy of "Positive Council"), Hasse B, Hirsch HH, Hoffmann M, Hösli I, Kahlert C, Kaiser L, Keiser O, Klimkait T, Kouyos R, Kovari H, Ledergerber B, Martinetti G, Martinez de Tejada B, Metzner K, Müller N, Nadal D, Nicca D, Pantaleo G, Rauch A (Chairman of the Scientific Board), Regenass S, Rickenbach M (Head of Data Centre), Rudin C (Chairman of the Mother & Child Substudy), Schöni-Affolter F, Schmid P, Schüpbach J, Speck R, Tarr P, Telenti A, Trkola A, Vernazza P, Weber R, Yerly S.

UK HIV Drug Resistance Database

Steering Committee: Celia Aitken (Gartnavel General Hospital, Glasgow); David Asboe, Anton Pozniak (Chelsea & Westminster Hospital, London); Patricia Cane (Public Health England, Porton Down); David Chadwick (South Tees Hospitals NHS Trust, Middlesbrough); Duncan Churchill (Brighton and Sussex University Hospitals NHS Trust); Duncan Clark (St Bartholomew's and The London NHS Trust); Simon Collins (HIV i-Base, London); Valerie Delpech (Centre for Infections, Public Health England); Samuel Douthwaite (Guy's and St. Thomas' NHS Foundation Trust, London); David Dunn, Esther Fearnhill, Kholoud Porter, Anna Tostevin, Ellen White (MRC Clinical Trials Unit at UCL, London); Christophe Fraser (Imperial College London); Anna Maria Geretti (Institute of Infection and Global Health, University of Liverpool); Antony Hale (Leeds Teaching Hospitals NHS Trust); Stéphane Hué (LSHTM, London); Steve Kaye (Imperial College, London); Paul Kellam (Wellcome Trust Sanger Institute & University College London Medical School); Linda Lazarus (Expert Advisory Group on AIDS Secretariat, Public Health England); Andrew Leigh-Brown (University of Edinburgh); Tamyo Mbisa (Virus Reference Department, Public Health England); Nicola Mackie (Imperial NHS Trust, London); Samuel Moses (King's College Hospital, London); Chloe Orkin (St. Bartholomew's Hospital, London); Eleni Nastouli, Deenan Pillay, Andrew Phillips, Caroline Sabin (University College London Medical School, London); Erasmus Smit (Public Health England, Birmingham Heartlands Hospital); Kate Templeton (Royal Infirmary of Edinburgh); Peter Tilston (Manchester Royal Infirmary); Daniel Webster (Royal Free NHS Trust, London); Ian Williams (Mortimer Market Centre, London); Hongyi Zhang (Addenbrooke's Hospital, Cambridge).

Coordinating Centre: MRC Clinical Trials Unit at UCL (Hannah Castro, David Dunn, Keith Fairbrother, Esther Fearnhill, Kholoud Porter, Anna Tostevin, Ellen White)
